# Complication of Orthodontic Treatment: A Case Report on Severe Apical Root Resorption (ARR) in a Patient with Turner Syndrome

**DOI:** 10.3390/children11030358

**Published:** 2024-03-18

**Authors:** Joanna Laskowska, Anna Paradowska-Stolarz, Lucía Miralles-Jordá, Dorota Schutty, Marcin Mikulewicz

**Affiliations:** 1 Division of Dentofacial Anomalies, Department of Maxillofacial Orthopedics and Orthodontics, Wroclaw Medical University, Krakowska 26, 50-425 Wroclaw, Poland; j.laskowska@umw.edu.pl (J.L.); dorota.kustrzycka@umw.edu.pl (D.S.); marcin.mikulewicz@umw.edu.pl (M.M.); 2 Department of Stomatology, Catholic University of Valencia, 46010 Valencia, Spain; lucia.miralles@ucv.es

**Keywords:** Turner syndrome, malocclusion, orthodontic treatment, root resorption

## Abstract

External apical root resorption in permanent teeth is a multifactorial process influenced by a variety of local and systemic factors. This report describes a case of multiple and severe apical root resorptions in a patient with Turner syndrome. The condition was discovered in a young female with Turner syndrome after 30 months of orthodontic treatment with fixed appliance. The purpose of this report is to present reports by other authors on the potential causes of the increased risk of tooth resorption in patients with Turner syndrome and to share insights derived from its course, highlighting the implications and lessons learned. Patients with Turner syndrome are not ideal candidates for orthodontic treatment. Prior to commencing orthodontic treatment, it is essential to carefully consider the potential benefits of the therapy compared to the risk associated with exacerbating root resorption. In the case of Turner syndrome patients, where there is an elevated risk of such complications, a thorough analysis should be conducted to determine whether the expected benefits of the treatment outweigh the potential hazards to the patient’s dental health.

## 1. Introduction

Turner syndrome is a rare genetic condition that has its origins in the total or partial lack of one of the X chromosomes. Because there is no chromosome Y, the syndrome refers to women only. Many cases are mosaics [1]. Turner syndrome is accompanied by reduced skeletal growth, retarded puberty, bone and skeletal deformities, and restricted joint motion, also associated with multiple osteochondromatosis. Those anomalies lead to a shortening of body stature and might be associated with premature osteoarthritis [1,2]. The limitations of height make women 20 cm shorter on average compared to women of the same age [3]. Patients are infertile due to ovarian dysgenesis. In addition, hypergonadotropic hypogonadism is observed. Girls and adult women may suffer from heart malformations and endocrine disorders, including diabetes mellitus of both types, osteoporosis, and autoimmune disorders. Due to the lack of typical changes, the diagnosis is often delayed and is either stated when growth hormone is applied to increase the height of an individual or in teenagers [1,3]. Due to gonadal dysgenesis and hormonal changes, hirsutism, virtiligo, and alopecia could be observed. Other features are a low posterior hairline, *pterygium colli* (short, webbed neck), and other small features such as low set ears and nail hypoplasia. The posture of the females are musculine, with wide-spaced, inverted nipples and a shield-shaped thorax [4].

Females with Turner syndrome have several occlusal problems, including severe malocclusions, and also dental anomalies. In the Japanese study [5], where patients with Turner syndrome were diagnosed orthodontically, bimaxillary retrognathia with deep skeletal bite was observed. The most popular maxillary abbreviations were class II malocclusions and a high-arched palate. The vaulted palate was also observed by other researchers [6]. Rarer extopic eruption of the teeth (including the first permanent molar) was observed, which could be caused by a discrepancy between bone size and its growth timing and tooth maturation.

The mesiodistal width of the teeth was also lower than in the non-syndromic population [5,6,7].

The eruption of permanent teeth is premature [5].

Apical root resorption is observed in 40% of cases of females with Turner syndrome during a 3.5 period of orthodontic treatment [8], but in this study the authors claim that there could be some limitation in ARR measurements due to the possible spatial projection errors observed on the panoramic X-rays. Patients with isochromosome Turner syndrome (45, X) were more prone to root resorption. The risk of root resorption is probably higher in females with Turner syndrome; therefore, frequent panoramic X-rays should be performed among these patients.

## 2. Materials and Methods

This case report describes an adolescent female patient with Turner syndrome (TS). The patient had shown genetic diagnosis, presenting a deletion of the short arm of chromosome X and mosaic pattern. The dental diagnosis included Angle’s class II subdivision with deep bite. 

### 2.1. Diagnosis and Aetiology

**Figure 1:**
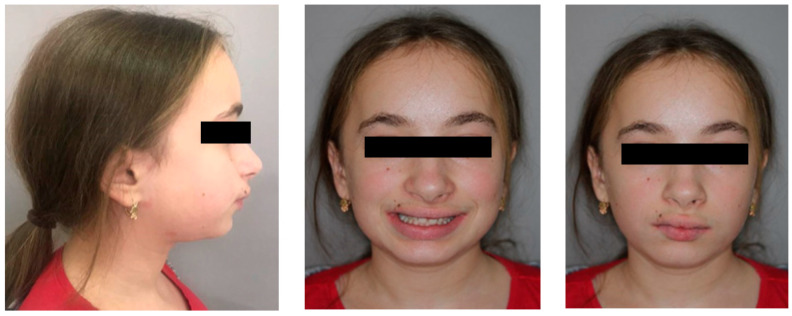
Pre-treatment extraoral photos.

A 14-year-old Caucasian patient from the Department of Paediatric Endocrinology and Diabetology was referred to the Division of Facial Abnormalities, Department *of Dentofacial Orthopaedics and Orthodontics* of the Wroclaw Medical University, Poland, seeking an orthodontic treatment and solutions to her aesthetic and functional disorders. Extraoral photos (Figure 1) and frontal examination revealed increased exposure of the incisor. The profile was convex and characterized by a mandibular retrusion and an increased lower facial height.

Clinical intraoral examination, performed in November of 2018, revealed class II subdivision 2, with lower midline deviation to the right of the upper midline. The clinicians also observed dental crossbite on a single tooth, 17, deep overbite, retrusion of the upper incisors, and slight spacing in the upper arch.

**Figure 2:**
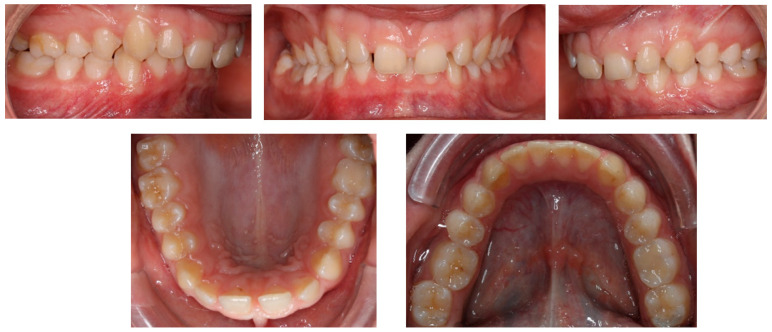
Pre-treatment intraoral photos (the **upper** line—right, mid, and left occlusion, the **lower** line—upper arch and lower arch forms).

A short periodontal examination, performed in our clinic in this kind of patient was performed. No need for further specialist examination was reported. The periodontal biotype and oral hygiene were both established as correct (Figure 2).

**Figure 3:**
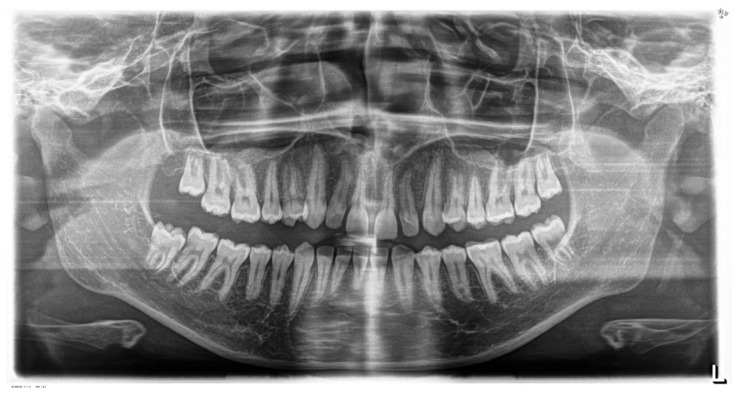
Pre-treatment panoramic radiograph.

Panoramic radiography, performed in 28 November 2018 (Densply Sirona, Charlotte, NC, USA, Orthophos SL 2D), revealed a full dentition and shortened tooth root length: 24, 25, 31, 32, 33, 34, 35, 41, 42, 43, 44, 45. The X-ray is presented in Figure 3.

**Figure 4:**
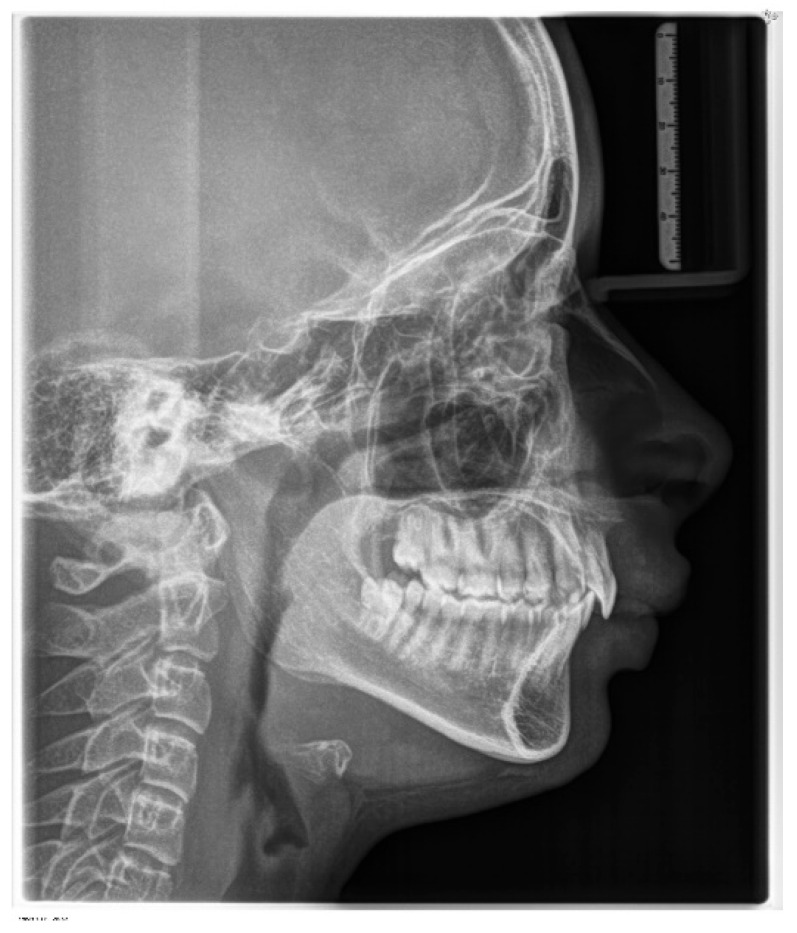
Pre-treatment lateral cephalometric radiograph.

According to latero-lateral teleradiography (Figure 4), she presented with an upper jaw retrusion combined with a lower jaw retrusion relative to the cranium (SNA = 81°, SNB = 73°, Wits appraisal of 7 mm) and a normal facial growth pattern (NS-Sar-ArGo-GoMe = 398.9°) with a normal mandibular plane angle (FMA = 25°, Gonial angle = 120°). Although the skeletal discrepancy was compensated by maxillary incisors’ retrusive inclination (upper incisor to SN = 89.48°, to NA = 1.55 mm) combined with normal mandibular incisors inclination (lower incisor to GoMe = 90°, to NB = 27°), an increased overjet of 15 mm was measured as reported in Table 1. Furthermore, a pretreatment lateral cephalometric radiograph revealed narrow airways. 

The clinical examination and anamnesis did not show any signs of bad oral habits.

### 2.2. Treatment Objectives 

The following treatment objectives were established and discussed with the patient and her parents: correction of the deep bite, improvement of the skeletal pattern, obtaining a class I molar and canine on both sides, flattening the Spee curve, and correction of the midline. Those tools were necessary to create a correct functional occlusion.

Due to the patient’s profile, the main objective was to correct the occlusion without penalising facial balance. The standard orthodontic procedures with the use of low orthodontic forces had been planned. The patient’s profile and the amount of class II malocclusion advised against extractive strategies. Therefore, we chose to align the teeth and open the deep bite as well as correct the unilateral class II malocclusion using the Twin Force Bite Corrector (TFBC) appliance, a fixed functional device placed unilaterally in combination with a comprehensive fixed appliances 0.022″ slot MBT brackets prescription (Ormco, Brea, CA, USA). As in every orthodontically treated patient, the prophylaxis with the use of a shaped orthodontic toothbrush, dental floss and an interdental brush was suggested. The patient was obliged to have a dental checkup every 6 months, with professional dental cleaning performed by a dentist or hygienist.

### 2.3. Treatment Progress

During the bonding in the upper arch, high-torque locks were used on the central and lateral incisors. Alignment was achieved using 0.016″ nickel–titanium (NiTi) heat-activated (HA) archwire. After 6 weeks, the levelling phase started in the upper arch with the application of 0.019″ × 0.025″ NiTi HA wires, and the lower arch brackets were also bonded. The levelling phase in the upper arch was completed with 0.019″ × 0.025″ stainless steel archwire (SS) less than five months after the bonding of the brackets. In the lower arch 0.021″ × 0.025″ stainless steel archwire (SS) was used. To begin unilateral full class II correction and improve the overbite, the Twin Force Bite Corrector (TFBC) appliance was placed approximately 7 weeks after the 0.021″ × 0.025″ SS archwire had been inserted into the mouth. The TFBC was placed on the wire in front of the band of the first maxillary molar and on the mandibular archwire, distally to the canine bracket, thus, creating a mesial force on the mandibular arch and a distal force on the maxillary arch. Once, after 3 months, the unilateral class II malocclusion was corrected, the Twin Force Bite Corrector appliance was removed and the unilateral class II elastics were placed on the right side to increase stability and maintain correction. 

To obtain the correct canine relationship, 18 months after bracket bonding, a miniscrew (Arcus 6 mm, AO) was placed in the interradicular area of teeth 15 and 16, additionally using a hook that allows the segment 13–23 to be moved to the right, toward the miniscrew. This stage of treatment lasted for one year, with interruptions resulting from the failure of the fixed appliance. 

After 2.5 years of orthodontic treatment, the patient was referred to the Department of Periodontology due to the calculus that covered more than one-third of the exposed tooth surface and the occlusal surface of the molars. During the appointment, cleaning and polishing of all teeth were performed.

**Figure 5:**
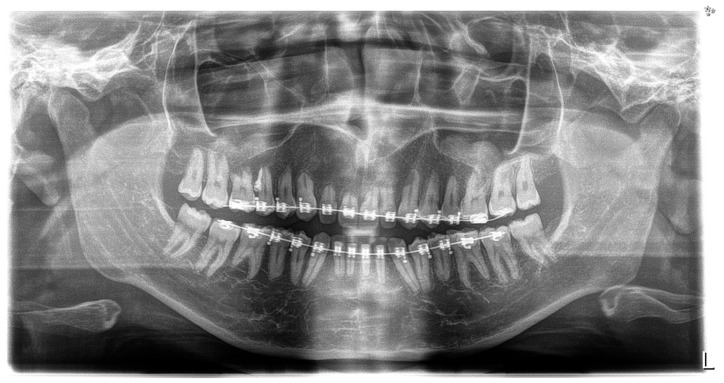
Panoramic radiograph at the end of the treatment. Severe root resorption was observed.

Orthodontic treatment with fixed appliances was continued for another 7 months. After 30 months of orthodontic treatment, the first pantomographic X-ray was taken during orthodontic treatment with fixed appliances. Panoramex showed advanced external resorption of the roots of the upper and lower arch teeth (Figure 5). The X-ray was taken using Orthophos SL 2D (Sirona) with exposure parameters of 69 kV, 12 mA through 14.1 s. The radiation rate was 127. According to this, the decision was made to immediately debond the brackets. After debonding grade 2 mobility of teeth 11 and 21, as part of retention, an upper and lower retention splint was used.

## 3. Results

**Figure 6:**
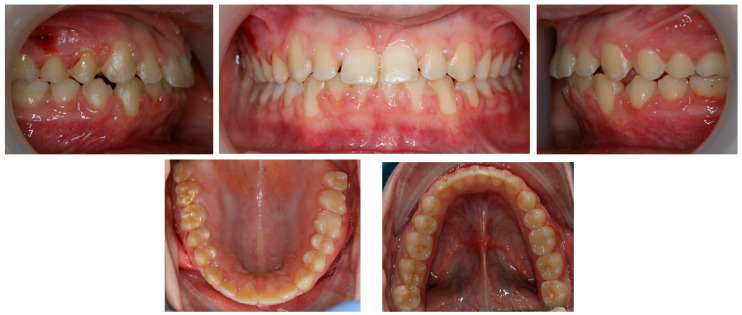
Post-treatment intraoral photos (the **upper** line—right, mid, and left occlusion, the **lower** line—upper arch and lower arch forms).

Intraoral examination reveals the achievement of many planned objectives, namely correction of the deep bite, improvement of molar and canine relation on both sides, flattening the curve of Spee, correction of the midlines, and spacing correction (Figure 6). 

**Figure 7:**
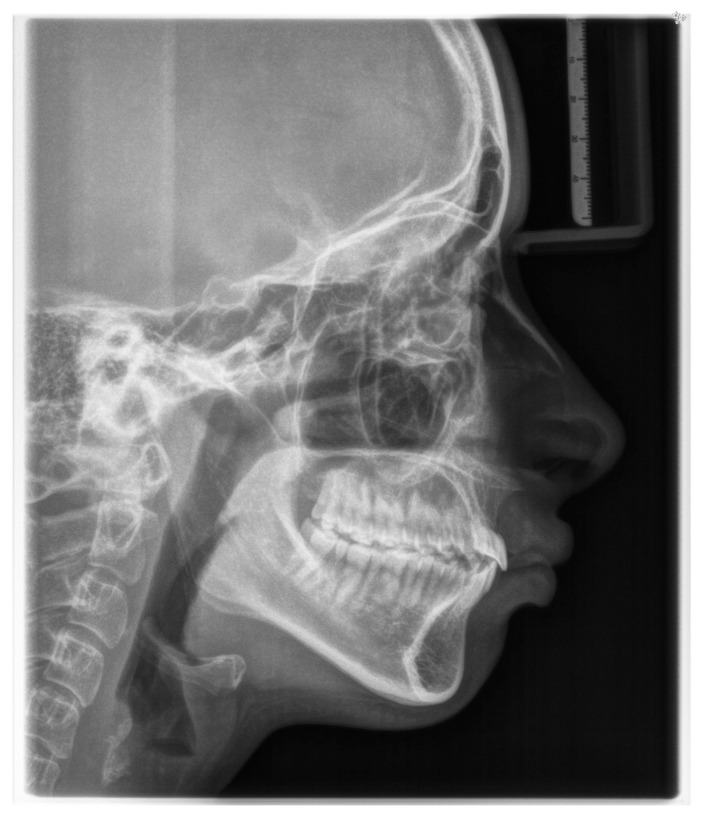
Post-treatment cephalometric radiograph.

**Figure 8:**
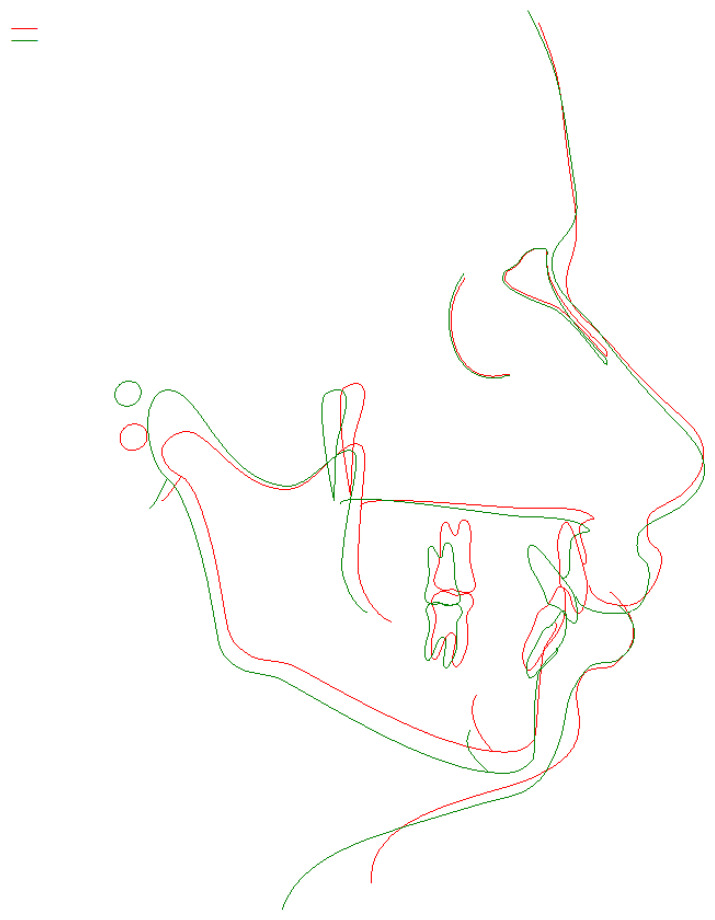
Pre- and post-treatment cephalometric superimposition. Green—post treatment, red—pre-treatment.

Post-treatment cephalometric X-ray was taken using Orthophos SL 2D (Sirona) with exposure parameters of 77 kV, 14 mA through 9.45 s. The radiation rate was 23. According to this, cephalometric indices and post-treatment latero-lateral teleradiography show the vertical improvement and the proclination of the lower incisors (Figure 7). The pre- and posttreatment cephalometric superimposition is shown in Figure 8.

**Figure 9:**
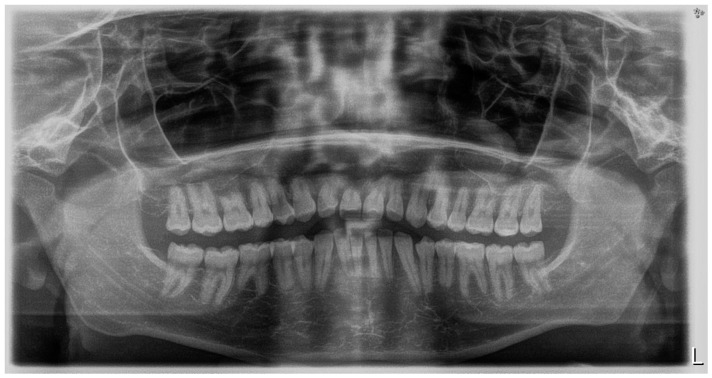
Panoramic radiograph one year after debonding.

**Figure 10:**
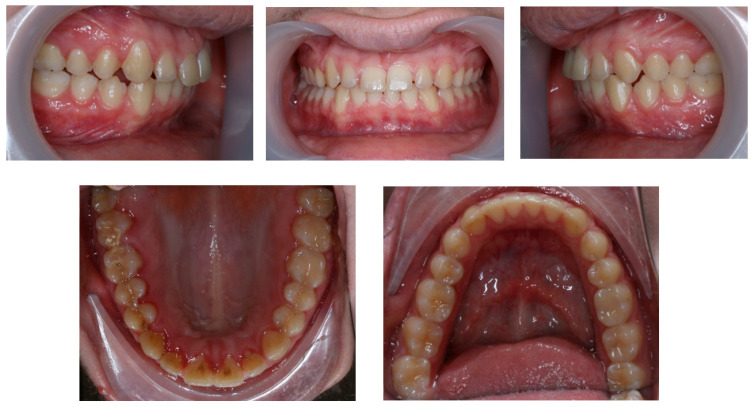
Intraoral photos after one year of debonding (the **upper** line—right, mid, and left occlusion, the **lower** line—upper arch and lower arch forms).

Post-treatment panoramic radiography was taken after one year of debonding and no evidence of additional apical root resorption was found. The mobility index of teeth 11 and 21, classified with Miller’s classification, had not changed. The pictures from the last checkup are presented in Figure 9 and Figure 10. 

## 4. Discussion

As in the data collected from the literature [6], the patient presented a class II deep bite, typical for women with Turner syndrome. She did not show any hypodontia, typical of syndromes with orofacial expression [9]. Our patient suffered from narrow airways, which might be typological for several types of malocculsions. Our patient suffers from a class II deep bite, which is also one of the factors that could cause narrowing of the airways [10,11]. As shown in other research [11], class II corrections result in a widening of the airways. This phenomenon was also present in our patient. 

The novel meta-analysis shows that regardless of the orthodontic method, the level of root resorption remains similar. The authors conclude that external apical root resorption (EARR) should be avoided because it is one of the most serious complications of orthodontic treatment [12]. The etiology of root resorption is not clear. Patients with a history of dental trauma, dental treatment, and systematic conditions influence the prevalence of apical root resorption [13]. The severe resorption observed in the patient could be a result of impaired metabolism and endocrinological problems, resulting from insufficient levels of growth hormone and estrogen, as reported among patients with Turner syndrome [14]. The main goal of the second hormone is to prevent bone resorption, so lowering its level often leads to consequences such as increased bone fragility, observed in osteoporosis [15]. This could also influence the resistance of the roots to damage and may lead to resorption, as observed in our patient. A possible way of preventing root resorption could be aligner treatment [16,17], because the treatment with aligners was proved to cause a smaller amount of apical root resorption when compared to the fixed appliance therapy. Although the amount of ARR is smaller when treatment with splints is applied, it is still present. The treatment with aligners was not popular at the time, when our patient began the active phase. To the best of the authors’ knowledge, there are no case presentations of this kind of treatment, and considering the lower bone density level in females with Turner syndrome [18], one could assume that the bone at the treatment site would behave differently.

Due to the potential risk of bone and teeth resorption, it would be suggested to have regular yearly X-ray examinations. The interesting clinical aspect would be the treatment with aligners as well, which are suggested to cause less resorptions than regular braces. Although all our patients sign the consent, in which they approve the potential risks of orthodontic treatment, in the cases where the tooth resorption is higher, additional talk on this topic should take place. 

The primary limitation of the study lies in the presentation of only a single patient. However, the observations of others and the discussed facts helped us form the conclusions.

## 5. Conclusions

Orthodontically, resorption could be triggered by high orthodontic forces and their type, the directions of exerted force and amount of movement (especially towards the apical part) [13]. While the authors acknowledge this potential risk, none of these were observed in our patient. Treatment was carried out using a low dose of forces. If too much apical movement is present, it could cause resorption of individual teeth, and severe resorption was observed throughout the dentition. Also, no true damage and large dental restorations were observed. 

In addition to the indisputable factor of the patient’s genetic predisposition, the potential cause of root resorption in the case described by the authors could be the duration of orthodontic treatment, which was 30 months. Researchers emphasize that patients whose orthodontic treatment with fixed appliances lasts longer experience significantly more grade 2 root resorption. Average treatment length for patients without root resorption is 1.5 years, and for the patients with severe root resorption, 2.3 years [19]. However, research suggests that root resorption may begin as early as six months into orthodontic treatment [20], indicating that the predispositions of the patients likely played a significant role in the majority of resorption cases.

To conclude, it’s worth noting that patients with Turner syndrome are not ideal candidates for orthodontic treatment. Prior to commencing orthodontic treatment, it is essential to carefully consider the potential benefits of the therapy compared to the risks associated with exacerbating root resorption. In the case of Turner syndrome patients, where there is an elevated risk of such complications, a thorough analysis should be conducted to determine whether the expected benefits of the treatment outweigh the potential hazards to the patient’s dental health.

## Figures and Tables

**Table 1:** Pre- and post-treatment cephalometric values.

	Pre-Treatment Value	Post-Treatment Value	Ref. Value
SNA (°)	81.03	80.69	82
SNB (°)	73.43	76.16	80
ANB (°)	7.61	4.52	2
Wits appraisal (mm)	6.73	2.17	−0.3
NS-Sar-ArGo-GoMe (°)	398.9	398.7	396
FMA (°)	25	24.79	25
Gonial angle (°)	120	125	125
U1–NA (mm)	1.55	3.32	4
U1–SN (°)	89.48	111.48	102
L1–NB (mm)	5.2	5.98	4
L1–NB (°)	26.99	31.77	25
L1–GoMe (°)	90.3	94.8	95
Nasolabial Angle	100.84	75.32	95
Lower Lip to E-Plane (mm)	1.66	3.65	−2
Upper Lip to E-Plane (mm)	2.55	1.97	−4.7
IMPA (°)	98.81	101.16	90
Overjet (mm)	4.87	3.98	2
Overbite (mm)	6.43	0.74	2

## Data Availability

Data are contained within the article.
